# Group-delivered interventions for lowering blood pressure in hypertension: a systematic review and meta-analysis

**DOI:** 10.3399/BJGP.2023.0585

**Published:** 2025-03-25

**Authors:** Sinéad TJ McDonagh, Charlotte Reburn, Jane R Smith, Christopher E Clark

**Affiliations:** Exeter Collaboration for Academic Primary Care, Department of Health and Community Sciences, Faculty of Health and Life Sciences, University of Exeter, Exeter.; Exeter Collaboration for Academic Primary Care, Department of Health and Community Sciences, Faculty of Health and Life Sciences, University of Exeter, Exeter.; Exeter Collaboration for Academic Primary Care, Department of Health and Community Sciences, Faculty of Health and Life Sciences, University of Exeter, Exeter.; Exeter Collaboration for Academic Primary Care, Department of Health and Community Sciences, Faculty of Health and Life Sciences, University of Exeter, Exeter.

**Keywords:** exercise, health education, hypertension, primary health care, systematic review, blood pressure

## Abstract

**Background:**

Hypertension is the leading modifiable cause of cardiovascular disease. Primary care management is predominantly individual and remains suboptimal. Interventions delivered to groups incorporate peer support and potentially offer efficient use of limited resources. Evidence for the benefits of group-delivered interventions in hypertension is unclear.

**Aim:**

To determine whether group-delivered hypertension interventions improve blood pressure (BP) outcomes compared to usual care (UC).

**Design and setting:**

Systematic review, meta-analyses, and meta-regression of randomised controlled trials in community, primary, or outpatient care settings.

**Method:**

MEDLINE, Embase, Cochrane CENTRAL, and CINAHL were searched from inception to 20 March 2024 for randomised controlled trials comparing group-delivered interventions to UC for adults with hypertension. Primary outcomes were changes in systolic and diastolic BP, achievement of study BP targets and medication adherence; quality was assessed using the Cochrane Risk of Bias 2 tool. Data were pooled according to intervention type using random effects meta-analyses; predictors of BP lowering were modelled with meta-regression.

**Results:**

Overall, 5326 citations were retrieved; 59 intervention groups (IGs) from 54 studies (13 976 participants) were included. Compared to UC, systolic BP reduced by 7.2 mmHg (95% confidence interval [CI] = 4.7 to 9.6; 23 IGs) following exercise, 4.8 mmHg (95% CI = 3.2 to 6.4; 26 IGs) following lifestyle education, and 3.6 mmHg (95% CI = 0.3 to 6.9; seven IGs) following psychotherapeutic interventions. Corresponding reductions in diastolic BP were 3.9 mmHg (95% CI = 2.6 to 5.2; 21 IGs), 2.9 mmHg (95% CI = 1.8 to 3.9; 24 IGs), and 1.2 mmHg (95% CI = −1.9 to 4.3; seven IGs). Achievement of target BP and medication adherence were infrequently reported, with equivocal findings (relative risks 1.1, 95% CI = 1.0 to 1.2, *P* = 0.02, 11 IGs and 1.0, 95% CI = 1.0 to 1.1, *P* = 0.60, seven IGs, respectively). In multivariable models, higher baseline BP and pre-existing cardiovascular morbidity were associated with greater BP reductions.

**Conclusion:**

Group-delivered interventions were effective at lowering BP for people with hypertension compared with UC; their feasibility and cost-effectiveness in primary care require further study.

## Introduction

Hypertension is the most common chronic condition encountered in primary care, and high blood pressure (BP) is the leading cause of cardiovascular disease worldwide.^1^ Lowering BP to below recommended thresholds, such as <140/90 mmHg as recommended by the National Institute for Health and Care Excellence (NICE), substantially reduces cardiovascular risk.^2^ Currently, hypertension is largely managed by individual patient consultations. However, these are a time-and resource-intensive method for delivering advice, particularly lifestyle and self-help advice, which is important for all people with hypertension, whether complicated with additional long-term conditions or not. Adherence to prescribed medications, and strategies to promote adherence, are generally inadequately delivered for good BP control.^3^ Alternative intervention approaches, particularly those involving non-pharmacological management, have been trialled using group interventions delivered to people with hypertension, for example, to learn about medications or exercise together. Such group-based interventions are attractive as they potentially offer more efficient use of limited time and professional resources. Furthermore, group interventions incorporate additional elements, such as peer support, that can enhance efforts to achieve and maintain lifestyle changes.^4^ Currently, it is unclear whether evidence supports adoption of group-delivered interventions for hypertension. Therefore, building on the authors’ ongoing Cochrane review,^5^ this systematic review was undertaken to determine whether, compared to usual care (UC), interventions delivered in groups improve BP outcomes for people with hypertension.

**Table:** How this fits in

Currently, hypertension is largely managed in primary care via individual patient consultations. Opportunities to offer effective lifestyle interventions and hypertension education within such consultations are restricted by resources and time. Many alternative intervention approaches, particularly those involving non-pharmacological management, have been trialled in group settings. Evidence to support the use of group-delivered interventions for hypertension is inconsistent, prompting this systematic review, meta-analysis, and meta-regression of current studies. This study found that, compared with usual care, group-delivered interventions were effective at reducing blood pressure in people with hypertension. The findings suggest that primary care at scale, such as within networks, could feasibly lower blood pressure using such group interventions, but cost-effectiveness has yet to be clearly demonstrated.

## Method

This review was conducted and reported according to the Preferred Reporting Items for Systematic Reviews and Meta-analyses (PRISMA) checklist.^6^ The study protocol was registered with PROSPERO (ID: CRD42019145126).

### Searching and review process

MEDLINE, Embase, Cochrane CENTRAL, and CINAHL were searched from their respective commencement dates to 20 March 2024 for randomised controlled trials comparing group-delivered interventions to UC for adults with hypertension. MeSH keywords were insufficiently specific for the review question. Therefore, a broad and simple search strategy was adopted following scoping searches to identify known studies (see Supplementary Appendix S1).^7^ Further studies were identified from authors’ archives and reference lists of review articles and included studies.

Two researchers independently screened titles and abstracts, assessed full texts for inclusion, undertook data extraction, and assessed study quality using the Cochrane risk of bias 2 (RoB 2) tool.^8^ Disagreements were discussed and adjudicated by a third author when required. The review process was managed using Covidence.

All authors undertook title, abstract, and full-text screening, and data extraction, which were reviewed by the fourth author before entry into the study dataset, with discussion between authors of any conflicts arising. The first and fourth authors undertook data analyses. The third and fourth authors advised on the study conduct, analysis, and interpretation of findings.

### Inclusion and exclusion criteria

Studies were eligible for inclusion if they were randomised controlled trials (RCTs) comparing group-delivered interventions in adults (aged ≥18 years) with treated or untreated hypertension, to a contemporaneous control group receiving either no intervention or UC. They were also eligible if interventions were delivered in community, primary, or outpatient care settings. Where participants had been selected based on a comorbid condition such as diabetes, the study was eligible if the entire sample had at least 50% prevalence of hypertension, or if results for a separate subgroup with hypertension were reported. Included studies were restricted to full-text English language publications and required to report at least one of the primary outcomes; pre-defined secondary outcomes were also extracted if reported (see Box 1).

**Box 1.** Primary and secondary outcomes

**Primary outcomes** Change in systolic and/or diastolic blood pressure from recruitment to follow-up. Systolic and/or diastolic blood pressure at recruitment and follow-up. Proportions achieving control of blood pressure (using stated study definitions of control). Proportion of patients taking anti-hypertensive medication at follow-up. **Secondary outcomes** Reporting of medication adherence, for example, use of Morisky scale. Reporting of harms, mortality, and/or cardiovascular morbidity. Health-related quality of life data. Costs and/or cost-effectiveness data for interventions.

Exclusion criteria were studies:

- with non-randomised study designs;
- with specific and specialised groups, for example, children or pregnancy;
- without UC or a no-intervention comparison group;
- recruiting inpatients or undertaken in secondary or tertiary care settings; and
- lacking ≥1 primary blood pressure outcomes, as defined in Box 1.

### Data extraction and study classification

Study-level data on participant characteristics, setting, type of intervention (exercise, lifestyle education, or psychotherapeutic), profession of intervention provider (nurse, pharmacist, dietitian, trainer, psychologist, or other), study definition of hypertension, and BP measurement method and device were extracted. Where interventions were multi-faceted, uncertainties over intervention type were agreed by author discussion according to defined rules (see Supplementary Appendix S2). For exercise-based interventions, intensity of exertion was classed as mild, moderate, or vigorous as defined by the Centers for Disease Control and Prevention according to the percentage of maximum predicted heart rate achieved.^9^ Since double blinding to allocation for any group intervention was not feasible, the RoB 2 domain *‘Bias due to deviations from intended intervention(s)’* was disregarded. Overall study quality was dichotomised as either low risk of bias (RoB) if the remaining four RoB 2 domains were all judged as low risk, or as high RoB if ≥1 of the four domains were judged as high risk. Where only presented graphically, BP data were extracted from suitable figures using WebPlotDigitizer (version 4.6).

### Statistical analysis

Aggregate study demographics were summarised as median and interquartile (IQR) ranges as appropriate. Where not reported, change in BP from baseline to follow-up at end of intervention was calculated using a matched pair approach.^10^ Where cluster RCTs displayed unit of analysis issues, adjustments were made using design effects and published intra-class correlation coefficients for systolic BP (SBP) and diastolic BP (DBP).^11^^,^^12^ For studies with >1 intervention group, UC participant numbers were split between comparisons to avoid double counting in analyses. Pooled estimates of weighted mean changes in BP outcomes were calculated in subgroups according to type of interventions and settings using *χ*^2^ tests. Random effects models were adopted a priori given anticipated heterogeneity between populations, settings, and interventions across included studies. Where single study results appeared at odds with the remainder, statistical heterogeneity was quantified using *I*^2^ statistics and explored using pre-specified subgroups and exploratory sensitivity analyses.

Univariable meta-regression analyses were undertaken to examine associations between study-level factors (type of intervention, mean age, percentage of females, mean absolute resting SBP and DBP, setting, BP measurement method [auscultatory or oscillometric], comorbidities [diabetes, chronic kidney disease, cardiovascular disease, and stroke], and change in BP). Factors suggesting univariable associations with BP outcomes (threshold *P*<0.25) were entered into multivariable models in a stepwise approach with age and sex included a priori.

Publication bias was assessed using funnel plots and quantified using the Egger’s test.^13^ Analyses were performed using Stata (version 17) and Risk-of-bias visualization (robvis).^14^

## Results

**Figure 1.**
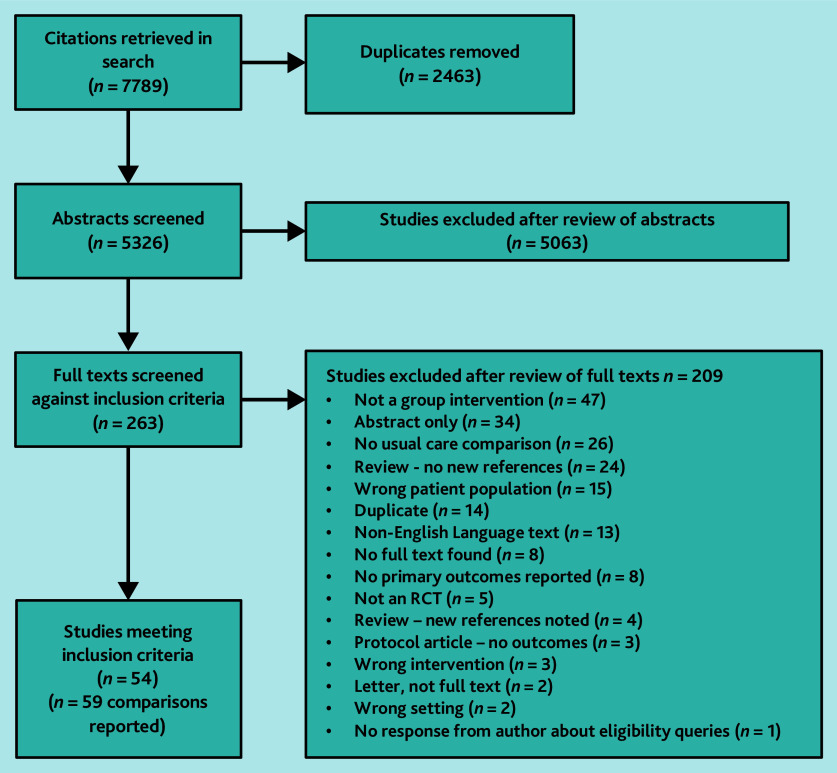
PRISMA chart. RCT = randomised controlled trial.

**Figure 2.**
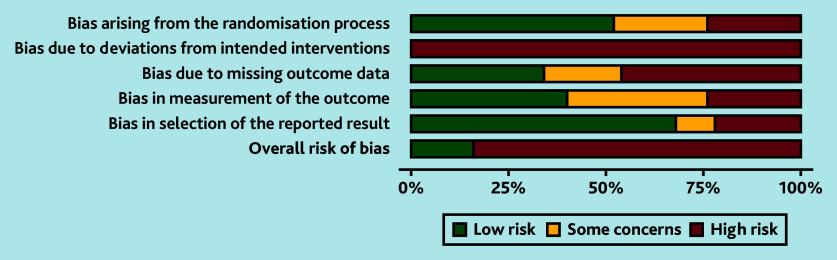
Risk of bias summary chart.

Searches identified 5326 unique citations. Out of the 263 full texts reviewed, 54 studies (containing 13 976 participants) met the inclusion criteria; four offered narrative data only and 50 studies, reporting on 59 interventions, contributed to the meta-analyses.^15^^–^^64^ Reasons for exclusion are summarised in Figure 1. Overall, study quality was poor with only seven studies (14%) rated as having a low overall RoB (Figure 2 and Supplementary Table S1).^20^^,^^21^^,^^29^^,^^41^^,^^48^^,^^57^^,^^62^

### Characteristics of study participants and interventions

Median publication year of included studies was 2015 (range 1988 to 2023). Included studies originated from the US (*n* = 14),^24^^,^^26^^,^^27^^,^^36^^,^^41^^,^^45^^,^^50^^,^^51^^,^^55^^,^^56^^,^^58^^,^^61^^–^^63^ China (*n* = 7),^20^^,^^38^^,^^39^^,^^46^^,^^49^^,^^53^^,^^57^ Iran,^15^^,^^21^^,^^22^^,^^43^^,^^64^ and other single countries across all continents except Australasia (see Supplementary Table S2).^16^^–^^19^^,^^23^^,^^25^^,^^28^^–^^35^^,^^37^^,^^40^^,^^42^^,^^44^^,^^47^^,^^48^^,^^52^^,^^54^^,^^59^^,^^60^ Seven studies were cluster RCTs^15^^,^^41^^,^^46^^,^^52^^,^^53^^,^^55^^,^^59^ and seven adjusted for clustering effects.^41^^,^^46^^,^^52^^,^^53^^,^^55^^,^^56^^,^^59^ Two studies shared a common UC arm.^16^^,^^17^ The majority of studies (30 out of 50; 60%) were conducted in community settings,^16^^–^^20^^,^^26^^,^^27^^,^^29^^–^^31^^,^^33^^,^^35^^–^^37^^,^^39^^,^^40^^,^^45^^,^^46^^,^^48^^,^^50^^–^^54^^,^^56^^,^^57^^,^^59^^,^^61^^,^^63^^,^^64^ 12 (24%) in primary care,^15^^,^^21^^,^^24^^,^^28^^,^^32^^,^^41^^,^^43^^,^^49^^,^^55^^,^^58^^,^^60^^,^^62^ and the remaining eight (16%) in secondary care outpatient settings.^22^^,^^23^^,^^25^^,^^34^^,^^38^^,^^42^^,^^44^^,^^47^

Of the 59 intervention groups (IGs), 24 (41%) were exercise-based,^16^^–^^19^^,^^27^^–^^40^^,^^64^ 28 (47%) lifestyle education (including elements of dietary and smoking advice, medication education, and adherence support),^15^^,^^20^^–^^24^^,^^26^^,^^41^^–^^59^ and seven (12%) psychotherapeutic (relaxation and stress management techniques).^25^^,^^60^^–^^63^ Thirteen of the 24 exercise IGs (54%) were classified as mild intensity,^18^^,^^19^^,^^29^^,^^32^^–^^34^^,^^38^^–^^40^^,^^64^ seven (29%) moderate,^16^^,^^27^^,^^28^^,^^31^^,^^33^^,^^35^^,^^36^ and four (17%) vigorous.^16^^,^^17^^,^^30^^,^^37^

Interventions delivered a median of 12 group sessions (interquartile range [IQR] 6–30) lasting 1 hour (IQR 0.8–1.5) over 3 months (IQR 2.5–6). Median group size was 14 (IQR 9–22), median participant age was 58 years (IQR 52–64), and median recruitment SBP was 141 mmHg (IQR 134–148) and DBP was 84 mmHg (IQR 82–89). Twelve IGs included females only^15^^–^^17^^,^^19^^,^^30^^,^^35^^,^^43^^,^^50^^,^^63^ and four males only;^23^^,^^25^^,^^31^^,^^37^ the remainder recruited both sexes.

Four eligible studies could not contribute to meta-analyses: one study of forgiveness training reported no change in mean BP; a Pranayama breathing intervention resulted in higher odds of attaining a 5 mmHg reduction in SBP (odds ratio [OR] 4.5; 95% confidence interval [CI] = 1.2 to 17.0); and one health education programme reported an improved composite score of BP plus medication use while the Multiple Risk Factor Intervention Trial reported greater reductions of DBP compared with UC.^65^^–^^68^

### Primary outcomes: systolic and diastolic BP

Overall, SBP and DBP reduced from recruitment to the end of interventions by 5.5 (95% CI = 4.2 to 6.8) and 3.1 (95% CI = 2.2 to 3.9) mmHg, respectively, compared to UC; these effects were consistent across community, primary, and outpatient care settings (see Supplementary Table S3). Outcomes did not differ between studies with low or high RoB (see Supplementary Table S4). Similar numbers of studies measured BP using auscultatory or automated sphygmomanometers; differences between measurement subgroups were accounted for by one outlying study that used an ambulatory monitor (see Supplementary Table S5).^29^ UC appeared to be enhanced by additional education for one-quarter of interventions;^20^^,^^22^^,^^28^^,^^35^^–^^37^^,^^39^^,^^41^^,^^46^^,^^48^^,^^52^^,^^53^^,^^56^^,^^62^ such enhancement did not affect the magnitude of intervention effects (see Supplementary Table S6). Funnel plots showed no evidence of small study bias (Egger’s tests: SBP change *P* = 0.23 and DBP change *P* = 0.97; Supplementary Figures S1 and S2).

### Subgroup analyses

**Figure 3.**
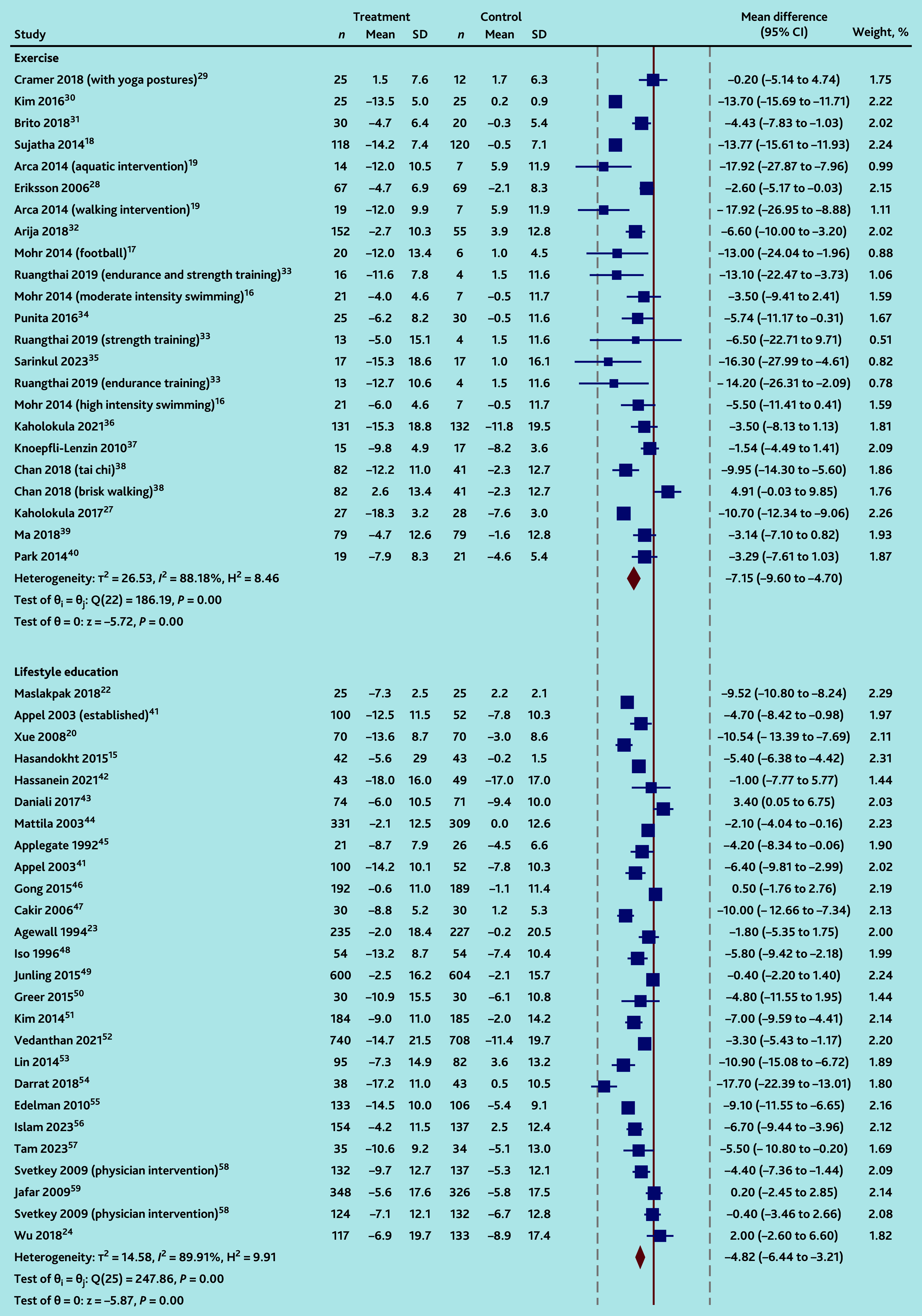

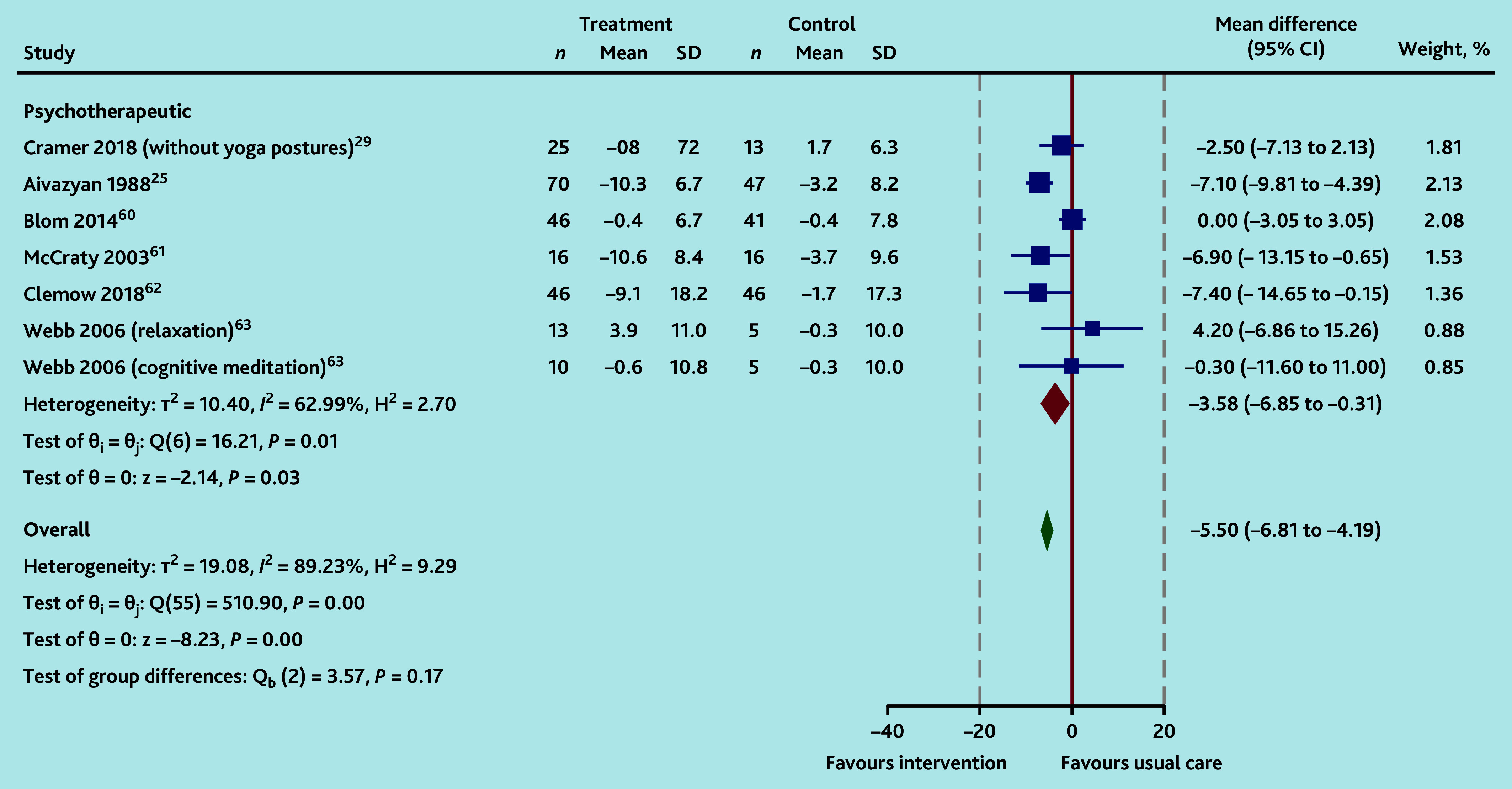
Random-effects DerSimonian-Laird model: changes in systolic blood pressure. SD = standard deviation. DASH = dietary approaches to stop hypertension.

Pooled BP reductions were significantly greater for each intervention type compared with UC, with significant between-study heterogeneity in all cases for SBP (*I*^2^ = 88% for 23 exercise IGs,^16^^–^^19^^,^^27^^–^^40^ 90% for 26 lifestyle education IGs,^15^^,^^20^^,^^22^^–^^24^^,^^41^^–^^59^ and 63% for seven psychotherapeutic IGs;^25^^,^^29^^,^^60^^–^^63^ *P* = 0.17 for subgroup differences, Figure 3) and DBP (*I*^2^ = 83% for 21 exercise IGs,^16^^–^^19^^,^^27^^–^^30^^,^^32^^–^^39^ 89% for 24 lifestyle education IGs,^15^^,^^20^^,^^22^^,^^23^^,^^41^^–^^51^^,^^53^^–^^58^ and 80% for seven psychotherapeutic IGs;^25^^,^^29^^,^^60^^–^^63^ *P* = 0.23 for subgroup differences, Supplementary Figure S3).

### Exercise interventions

Mean SBP and DBP reductions were −7.1 (95% CI = −9.6 to −4.7; 23 IGs^16^^–^^19^^,^^27^^–^^40^) and −3.9 (95% CI = −5.2 to −2.6; 21 IGs^16^^–^^19^^,^^27^^–^^30^^,^^32^^–^^39^) mmHg, respectively, for exercise interventions compared to UC. Weighted mean reductions differed by type of exercise (*χ*^2^ for differences between exercise groups SBP and DBP *P*<0.001; Supplementary Figures S4 and S5), with pooled findings based on small numbers of studies for each type as follows: SBP and DBP were reduced more with two aquatic exercise interventions^19^^,^^30^ compared to UC (−13.9 mmHg, 95% CI = −15.8 to −11.9, *I*^2^ = 0% and −6.1 mmHg, 95% CI = −6.7 to −5.5, *I*^2^ = 0%, respectively). Two swimming interventions^16^ lowered SBP compared to UC with low heterogeneity (−4.5 mmHg, 95% CI = −8.7 to −0.3, *I*^2^ = 0%); differences in DBP were uncertain (−1.6 mmHg, 95% CI = −6.1 to 3.0, *I*^2^ = 0%).

Consistent DBP, but uncertain SBP reductions compared to UC, were observed following three endurance training interventions:^28^^,^^33^ SBP −8.7 mmHg (95% CI = −17.4 to 0.1, *I*^2^ = 73%) and DBP −2.5 mmHg (95% CI = −3.8 to −1.2, *I*^2^ = 0%); two football training interventions:^17^^,^^37^ SBP −6.0 mmHg (95% CI = −16.9 to 5.0, *I*^2^ = 74%) and DBP −5.4 mmHg (95% CI = −8.1 to −2.7, *I*^2^ = 0%); and two tai chi interventions:^38^^,^^39^ SBP −6.5 mmHg (95% CI = −13.2 to 0.2, *I*^2^ = 81%) and DBP −5.2 mmHg (95% CI = −6.8 to −3.6, *I*^2^ = 0%).

SBP change was inconsistent following two dance interventions^27^^,^^36^ compared to UC (−7.4 mmHg, 95% CI = −14.5 to −0.4, *I*^2^ = 88%); but DBP was reduced (−2.8, 95% CI = −3.7 to −1.9, *I*^2^ = 0%).

Reductions in SBP and DBP with group-based yoga:^18^^,^^29^^,^^34^ −6.8 mmHg (95% CI = −15.8 to 2.2, three IGs, *I*^2^ = 93%) and −3.5 mmHg (95% CI = −8.8 to 1.8; four IGs; *I*^2^ = 90%), respectively, and walking interventions:^19^^,^^32^^,^^38^ −5.9 mmHg (95% CI = −16.5 to 4.6; three IGs; *I*^2^ = 92%) and 1.1 mmHg (95% CI = −5.3 to 3.1; three IGs; *I*^2^ = 74%), respectively, were uncertain, with significant heterogeneity between studies. Sensitivity analyses, excluding single outlying strongly positive studies, explained diastolic heterogeneity between the three remaining yoga studies (residual *I*^2^ = 0%),^18^^,^^29^^,^^34^ and substantially reduced it (residual *I*^2^ = 55%) between the two remaining walking studies;^32^^,^^38^ though pooled results remained uncertain (see Supplementary Figure S6).

**Figure 4.**
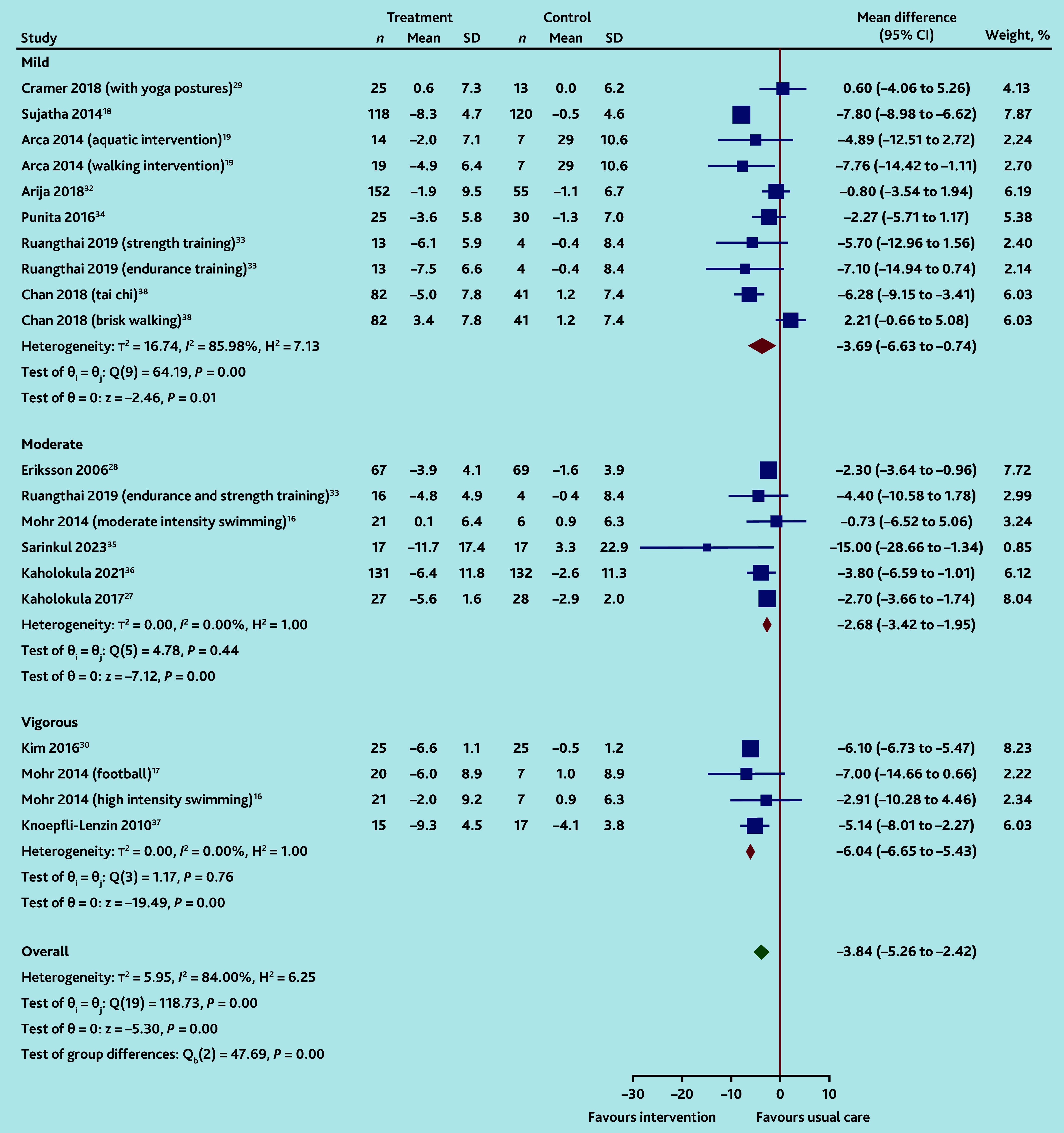
Random-effects DerSimonian-Laird model: changes in diastolic blood pressure by intensity of exercise. SD = standard deviation.

Reductions in DBP but not SBP, compared to UC, were greater with vigorous exercise interventions than with moderate or mild intensity. For SBP changes there was significant heterogeneity across all interventions (vigorous: −8.1 mmHg, 95% CI = −15.9 to −0.3, four IGs,^16^^,^^17^^,^^30^^,^^37^ *I*^2^ = 94%; moderate: −6.7 mmHg, 95% CI = −10.5 to −2.8, seven IGs,^16^^,^^27^^,^^28^^,^^31^^,^^33^^,^^35^^,^^36^ *I*^2^ = 85%; and mild: −7.7 mmHg (95% CI = −12.1 to −3.3, 11 IGs,^18^^,^^19^^,^^29^^,^^32^^–^^34^^,^^38^^,^^40^ *I*^2^ = 88%; *χ*^2^ for differences between subgroups *P* = 0.91; Supplementary Figure S7). For DBP changes, heterogeneity was only evident for mild intensity exercises (vigorous: −6.0 mmHg, 95% CI = −6.5 to −5.4, four IGs,^16^^,^^17^^,^^30^^,^^37^ *I*^2^ = 0%; moderate: −2.7 mmHg, 95% CI = −3.4 to −1.9, six IGs,^16^^,^^27^^,^^28^^,^^33^^,^^35^^,^^36^ *I*^2^ = 0%; and mild: −3.7 mmHg, 95% CI = −6.6 to −0.7, 10 IGs,^18^^,^^19^^,^^29^^,^^32^^–^^34^^,^^38^ *I*^2^ = 86%; *χ*^2^ for differences between subgroups *P*<0.001; Figure 4).

### Lifestyle educational interventions

SBP and DBP reductions were larger following group-based lifestyle education interventions than UC (−4.8 mmHg, 95% CI = −6.4 to −3.2, 26 IGs,^15^^,^^20^^,^^22^^–^^24^^,^^41^^–^^59^ *I*^2^ = 90%, Supplementary Figure S8; and −2.9 mmHg, 95% CI = −3.0 to −1.8, 24 IGs,^15^^,^^22^^–^^24^^,^^42^^–^^56^^,^^58^^,^^59^ *I*^2^ = 89%, Supplementary Figure S3, respectively). Between-study heterogeneity was partially explained for SBP but not DBP differences by RoB: SBP and DBP reductions for low RoB studies compared to UC were −6.9 mmHg (95% CI = −9.2 to −4.5, five IGs,^20^^,^^41^^,^^48^^,^^57^ *I*^2^ = 51%) and −2.5 mmHg (95% CI = −6.0 to 1.0, five IGs,^20^^,^^41^^,^^48^^,^^57^ *I*^2^ = 91%), respectively, in contrast to −4.4 mmHg (95% CI = −6.2 to −2.6, 21 IGs,^15^^,^^22^^–^^24^^,^^42^^–^^47^^,^^49^^–^^56^^,^^58^^,^^59^ *I*^2^ = 91%) and −2.9 mmHg (95% CI = −4.0 to −1.8, 19 IGs,^15^^,^^22^^,^^23^^,^^42^^–^^47^^,^^49^^–^^56^^,^^58^^,^^59^ *I*^2^ = 89%), respectively, for high RoB studies (*χ*^2^ for differences between RoB subgroups: SBP *P* = 0.11, Supplementary Figure S8, and DBP *P* = 0.85, Supplementary Figure S9). Sensitivity analysis excluding one highly positive 5-week intervention^20^ explained SBP and substantially reduced DBP between-study heterogeneity for low RoB studies. For the remaining four study groups, pooled differences in SBP and DBP were −5.7 mmHg (95% CI = −7.6 to −3.7, residual *I*^2^ = 0%, Supplementary Figure S10) and −1.2 mmHg (95% CI = −3.3 to 0.9, residual *I*^2^ = 64%, Supplementary Figure S11), respectively.

### Psychotherapeutic interventions

Compared to UC, pooled SBP was −3.6 mmHg (95% CI = −6.9 to −0.3, *I*^2^ = 63%, seven IGs^25^^,^^29^^,^^60^^–^^63^) lower but DBP effects were uncertain (−1.2 mmHg, 95% CI = −4.3 to 1.9, *I*^2^ = 80%, seven IGs^25^^,^^29^^,^^60^^–^^63^). Heterogeneity between IGs was explained by RoB: for two IGs^29^^,^^62^ at low RoB BP reduction was uncertain compared to UC (SBP −4.1 mmHg, 95% CI = −8.6 to 0.4, *I*^2^ = 20%, Supplementary Figure S12; DBP −2.2 mmHg, 95% CI = −5.4 to 0.9, *I*^2^ = 0%, Supplementary Figure S13).

### Ambulatory BP

Four comparisons (two from one study at low RoB) reported changes in 24-hour ambulatory BP,^29^^,^^60^^,^^64^ finding no difference between interventions and UC in SBP (2.3 mmHg, 95% CI = −1.2 to 5.8, *I*^2^ = 56%) or DBP (1.2 mmHg, 95% CI = −1.4 to 3.7, *I*^2^ = 54%).

### Blood pressure target achievement

Eleven comparisons (10 lifestyle education and one exercise IG^23^^,^^26^^,^^32^^,^^42^^,^^51^^,^^54^^–^^56^^,^^58^^,^^59^), all at high RoB, reported attainment of study BP targets of <140/90 mmHg in nine studies^26^^,^^32^^,^^51^^,^^54^^–^^56^^,^^58^^,^^59^ (one also targeting 130/80 mmHg in the presence of diabetes^30^), <140/80 mmHg in another,^42^ and a DBP target of <90 mmHg for one other.^23^ Pooled relative risk (RR) for achievement of study targets was 1.1 (95% CI = 1.0 to 1.2, *P* = 0.02, Supplementary Figure S14), with low heterogeneity (*I*^2^ = 28%), unaffected by intervention type.

### Antihypertensive medication use

Seven studies^20^^,^^47^^,^^49^^,^^53^^–^^55^^,^^59^ (one at low RoB^20^) reported rates of medication use following lifestyle education interventions. Pooled results showed no clear overall impact with low heterogeneity between studies (RR 1.0, 95% CI = 1.0 to 1.1, *I*^2^ = 0%, Supplementary Figure S15).

### Secondary outcomes

Limited data were available for specified secondary outcomes, precluding any quantitative synthesis. One lifestyle education intervention reported improvement in the validated 8-item Morisky medication adherence score (mean score pre-intervention 4.2 [SD 0.8] and post-intervention 6.7 [SD 0.5], *P*<0.001 for change); UC unchanged (pre-intervention 3.8 [SD 1.1] and post-intervention 3.7 (SD 0.1), *P* = 0.56 for change).^21^ Another lifestyle education intervention reported improved medication adherence assessed using the Hill–Bone compliance to high BP therapy scale.^22^

One trial reported lower incidence of stroke after 3 years following a multiple risk factor lifestyle education intervention (2.0%) compared to UC (6.7%) (*P* = 0.017).^23^ Only one lifestyle education trial examined costs, from a Veteran’s Health Administration perspective. Overall costs were no different between arms after the 13-month intervention, with lower outpatient costs balanced by increased medication costs for the intervention compared to UC.^24^

Health-related quality of life was assessed using different scales in five RCTs (see Supplementary Table S7):^20^^,^^24^^–^^27^ individual interventions achieved improved ratings on the Soviet Quality of Life score,^25^ and an improved score for perceived benefits of controlling BP (though other domains did not improve).^26^ Two RCTs used the Short-Form Health Survey (SF)-12 instrument; one reported greater physical and mental domain improvements and the other showed no change.^20^^,^^27^ Another study found no improvement using the SF-36.^24^

### Meta-regression

Univariable meta-regression of SBP and DBP outcomes showed that higher baseline BPs, enhanced UC, and inclusion of greater proportions with pre-existing cardiovascular comorbidities or diabetes were associated with greater BP differences between intervention and UC groups. Size of intervention groups (for SBP only) and lower baseline use of antihypertensive medications (for DBP only) were also associated with greater differences. Greater exercise intensity was associated with greater BP differences following exercise interventions (see Supplementary Table S8).

**Figure 5.**
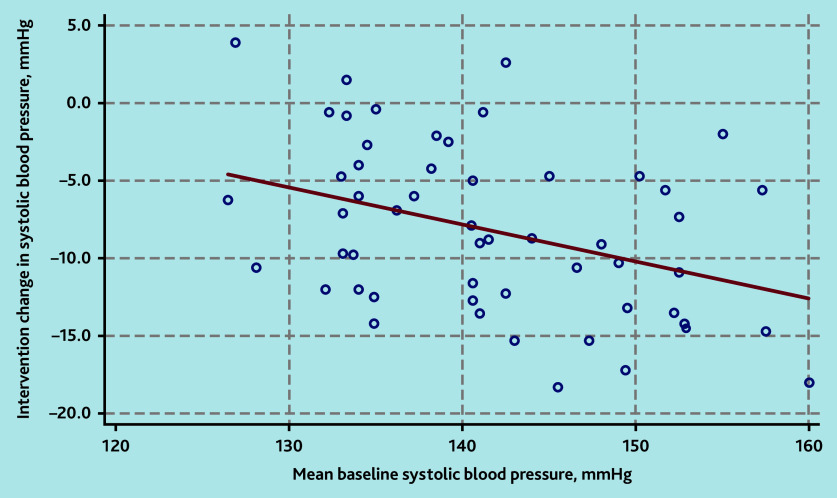
Association of higher baseline systolic blood pressure with greater systolic blood pressure reduction on intervention.

In multivariable models, higher baseline SBP (*P* = 0.021, Figure 5) remained as a predictor of greater reductions in SBP following interventions (see Supplementary Table S9). Pre-existing cardiovascular morbidity, baseline medication use, and female sex predicted greater reductions in DBP (see Supplementary Table S10 and Supplementary Figure S16).

## Discussion

### Summary

To the authors’ knowledge, this is the first review to synthesise evidence for group-based interventions in hypertension. Pooled findings from 50 RCTs showed greater reductions in SBP and DBP following exercise, lifestyle education, or psychotherapeutic interventions compared to UC in people with hypertension, delivered across a broad range of care settings. Different exercise interventions varied in effect, with greater exercise intensity being associated with greater BP reductions. Existing cardiovascular morbidity and higher baseline SBP were associated with greater BP reductions for group-based interventions compared to UC.

### Strengths and limitations

Broad search terms identified as many relevant studies as possible. Data extraction was restricted to English language publications and grey literature was not explored. However, such omissions generally show limited impacts on review findings where, as here, a substantial body of published evidence is retrieved.^69^ No evidence of small study or publication bias was found; however, few studies were judged to be low RoB using the RoB 2 tool.^8^ This tool is appropriate for the assessment of RCTs, though the types of interventions included here cannot achieve participant blinding or facilitators to allocation, rendering assessment of this RoB domain redundant. There was significant statistical heterogeneity between studies that could only be partially explained (for example, using exercise intensity) by subgroup and sensitivity analyses. Residual heterogeneity between studies probably reflects the diversity of interventions and populations studied. Given the small number of studies in some planned subgroups, the authors are cautious over the strength of the present study findings. The co-primary outcomes of BP target achievement and antihypertensive medication use, and all secondary outcomes, were reported by few studies, preventing firm conclusions being drawn. Lifestyle interventions consistently recruited predominantly urban-based White females, with underrepresentation of ethnic minorities, males, and rural-based participants, limiting the ability to generalise findings for such interventions.^70^

### Comparison with existing literature

Comprehensive interventions to lower BP include lifestyle modification targeting smoking, diet, exercise, and medicines management.^71^ These interventions are more effective when led by nurses and pharmacists in comparison to UC, particularly when they include changes in medication.^72^ International hypertension guidelines encourage this, but without detail on how to implement such interventions.^2^^,^^73^ Non-pharmacological interventions are beneficial;^72^^,^^74^ however, many are intensive and their implementation would exceed the available resources of existing healthcare systems. The present review suggests that such interventions could be effective in lowering BP if delivered to groups of patients in primary, community, and outpatient settings. Exercise has a role in lowering BP with evidence for aerobic exercise, endurance training, or dynamic and isometric resistance training.^75^ Existing review evidence supports the association of greater BP reductions with increasing intensity of exercise.^76^

Qigong has been shown to be effective in reducing BP, but no more so than other modes of exercise.^76^ NICE recommendations for relaxation therapies in hypertension were removed from the 2019 guidelines due to lack of evidence for effectiveness, with a call for further research.^2^ Previous reviews have suggested that meditation produces modest reductions in BP in general populations, but few studies have focused on people with hypertension or on group delivery.^77^^,^^78^ This review found no new evidence to support such interventions in group settings.

Though quality-of-life measures showed no changes compared to UC, generic quality-of-life tools are often non-discriminatory in hypertension owing to ceiling effects; condition-specific outcome measures may be more sensitive.^79^

### Implications for research and practice

Hypertension affects the majority of people aged >65 years.^1^ In contrast to Diabetes Education and Self-Management for Ongoing and Newly Diagnosed (DESMOND) diabetes or pulmonary rehabilitation for chronic obstructive pulmonary disease (COPD),^80^^,^^81^ the much commoner diagnosis of hypertension does not routinely offer access to any comparable group-based structured intervention.^2^ NHS health checks diagnose new hypertension in 2.5% of attendees.^82^ Median group size in this review was 14, which approximates to the annual number of new hypertension diagnoses for two small general practices.^83^ Thus, a group-based intervention delivered regularly at English primary care-network level could provide such a programme at appropriate scale for its population, offering a resource for health and wellbeing coaches, or other members of the primary healthcare team. The greatest BP lowering was seen with higher baseline BPs, analogous to uncontrolled hypertension, and with pre-existing cardiovascular morbidity. This suggests that such interventions could most usefully be targeted at people soon after hypertension is diagnosed; a critical time period when intensive intervention can achieve significant BP and future mortality reductions.^84^^,^^85^ The present study findings suggest that multimorbidity, which exists for most people with hypertension,^86^ should encourage rather than preclude people from enrolment. Typical SBP reductions per full-dose antihypertensive drug are 10–15 mmHg, thus these interventions have the potential to reduce medication intensification, or classification as resistant hypertension, for a proportion of patients.^87^

No cost-effectiveness data applicable to a UK perspective were found. In general, NICE considers drug initiation for people with a 10% predicted 10-year cardiovascular risk to be cost effective.^2^ However, acquisition costs are low for generic antihypertensive drugs. Health economic analyses of non-pharmaceutical interventions in hypertension are uncommon; those in existence provide only equivocal, low-quality evidence.^88^ Without full economic analyses, it cannot be assumed that the demonstrated beneficial BP effects of group-based interventions can be achieved at acceptable costs. Therefore, further carefully designed RCTs of group-based hypertension interventions with formal cost-effectiveness analyses are required.

## Data

All studies included or referenced in this study are publicly available.
